# Adaptive laboratory evolution triggers pathogen-dependent broad-spectrum antimicrobial potency in *Streptomyces*

**DOI:** 10.1186/s43141-021-00283-3

**Published:** 2022-01-03

**Authors:** Dharmesh Harwani, Jyotsna Begani, Sweta Barupal, Jyoti Lakhani

**Affiliations:** Department of Computer Science, Maharaja Ganga Singh University, Bikaner, Rajasthan India

**Keywords:** *Streptomyces*, Adaptive evolution, Induction, Antimicrobials, Mutants, RAPD

## Abstract

**Background:**

In the present study, adaptive laboratory evolution was used to stimulate antibiotic production in a *Streptomyces* strain JB140 (wild-type) exhibiting very little antimicrobial activity against bacterial pathogens. The seven different competition experiments utilized three serial passages (3 cycles of adaptation-selection of 15 days each) in which *Streptomyces* strain (wild-type) was challenged repeatedly to one (bi-culture) or two (tri-culture) or three (quadri-culture) target pathogens. The study demonstrates a simple laboratory model to study the adaptive potential of evolved phenotypes and genotypes in *Streptomyces* to induce antibiotic production.

**Results:**

Competition experiments resulted in the evolution of the wild-type *Streptomyces* strain JB140 into the seven unique mutant phenotypes that acquired the ability to constitutively exhibit increased antimicrobial activity against three bacterial pathogens *Salmonella* Typhi (NCIM 2051), *Staphylococcus aureus* (NCIM 2079), and *Proteus vulgaris* (NCIM 2027). The mutant phenotypes not only effectively inhibited the growth of the tested pathogens but were also observed to exhibit improved antimicrobial responses against one clinical multidrug-resistant (MDR) uropathogenic *Escherichia coli* (UPEC 1021) isolate. In contrast to the adaptively evolved mutants, only a weak antimicrobial activity was detected in the wild-type parental strain. To get molecular evidence of evolution, RAPD profiles of the wild-type *Streptomyces* and its evolved mutants were compared which revealed significant polymorphism among them.

**Conclusion:**

The competition-based adaptive laboratory evolution method can constitute a platform for evolutionary engineering to select improved phenotypes (mutants) with increased antibacterial profiles against targeted pathogens.

**Supplementary Information:**

The online version contains supplementary material available at 10.1186/s43141-021-00283-3.

## Background

The discovery of antibiotics by Alexander Flemming in 1929 revolutionized the treatment of diseases caused by microbial pathogens [1]. At present times, the numbers of infections caused by MDR *Staphylococcus aureus*, *Acinetobacter baumannii*, *Klebsiella pneumoniae*, *Pseudomonas aeruginosa*, *Mycobacterium tuberculosis*, etc., have been found to pose serious threats to human health. Consequently, efforts are being made globally to develop new antibiotic compounds to prevent these deadly pathogens [2]. The majority of antibiotics are natural products or their semi-synthetic derivatives that originated from the genus *Streptomyces* in the order *Actinomycetales* [3, 4], which are known to be distributed mainly in soil and marine sediments. The clinically important antibiotics produced by *Streptomyces* species include tetracyclines, aminoglycosides (e.g., streptomycin, neomycin, kanamycin), macrolides (e.g., erythromycin), chloramphenicol, and rifamycins [5, 6]. Many new antibiotics were isolated from actinomycetes between the late 1940s and the late 1960s, a period of the “Golden Age” of antibiotic discovery. After that, the rate of discovery gradually declined due to the frequent re-discovery of already known chemical compounds (secondary metabolites). However, the rapid increase in the recent genome sequence information suggests that this source is still not yet exhausted [7–12].

In the present paper, we hypothesized that a strain of *Streptomyces* that exhibits very little antimicrobial activity could undergo evolution, if challenged against a bacterial pathogen. Repeated exposure to a pathogen could lead to the production of antimicrobials that are usually not produced by its parental strain. It was also hypothesized that challenging the wild-type strain with more than one pathogen in adaptive evolution may elicit a different response. In other studies, co-cultures of two different organisms have been observed to improve the production of antimicrobial molecules [13–17]. Importantly, these experiments have not used adaptive-selection cycles as these used in the present study. The co-culture experiments involve the cultivation of two (or more) microbes (interspecies interactions) in the same closed and restricted environment for a certain period. The supernatant from these mixed cultures and mono-culture (control) is then analyzed for their bio-activity or no bio-activity. In contrast to this approach, in the laboratory evolution experiments described in the present study, the wild-type *Streptomyces* strain JB140 is co-cultured with different bacterial pathogens in the bi-, tri-, and quadri-culture assays. After isolation and purification of the *Streptomyces* phenotypes from the first cycle, these phenotypes were repeatedly exposed (three successive cycles of adaptation-selection of 15 days each) to the bacterial pathogens until the bio-activity was detected.

## Methods

### Bacterial strains

The present research utilized a *Streptomyces* strain JB140 (wild-type) exhibiting very little antimicrobial activity, its seven mutants, namely JB140^*Pv*^, JB140^*St*^, JB140^*Sa*^, JB140^*Sa,St*^, JB140^*Sa,Pv*^, JB140^*St,Pv*^, and JB140^*Sa,St,Pv*^ purified after the third (III) cycle of serial adaptation and selection as mentioned below in the results section, three bacterial pathogens *Salmonella* Typhi (NCIM 2051), *Staphylococcus aureus* (NCIM 2079), *Proteus vulgaris* (NCIM 2027), and one clinical multidrug-resistant (MDR) uropathogenic *Escherichia coli* (UPEC 1021) isolate.

### Selective isolation, maintenance, and characterization

The isolate JB140 was recovered from the soil sample collected at Ganganagar (29.9038°N, 73.8772°E) region falling in the arid Thar desert in the Indian state of Rajasthan. After selective isolation, using the modified actinomycete selective (MAS) agar medium, the pure culture of the isolate was stored at – 40 °C using 20% (w/v) sterile glycerol until further use. The isolate JB140 was further subjected to its morphological studies using the bright field and scanning electron microscopy (SEM). The sample for SEM analysis was prepared according to the protocol described by Srinivasan et al. [18]. Phenotypic characterization of the isolate was performed by growing it on ISP (International *Streptomyces* Project) media as described by Shirling and Gottlieb [19]. Taxonomic identification of the isolate JB140 was performed using 16SrRNA gene sequence analysis which is described below in the methods section.

### Cultivation and evaluation of antimicrobial activity of the isolate JB140

After phenotypic characterization, the isolate was screened for its antibacterial activity against bacterial pathogens in primary and secondary screening using agar plug [20, 21] and agar well diffusion [22, 23] methods, respectively. For small-scale cultivation, the isolate was cultured in a 1L flask containing 200 mL of ISP2 medium. The cultivation was carried out at 37 °C for 10 days at an agitation rate of 120 rpm. The growth was monitored by measuring optical density at 600 nm for 15 days at 2 days intervals. After cultivation, the broth was extracted with ethyl acetate. The extract was concentrated to dryness and the residue crude extract was dissolved in methanol and stored at 4 ^o^C for further use. The methanolic extracts were used to evaluate antimicrobial activity in the agar well diffusion method. Antimicrobial activity at different time points was also monitored during the time course of cultivation. The antimicrobial activity was assayed against three type strains *Salmonella* Typhi (NCIM 2051), *Staphylococcus aureus* (NCIM 2079), *Proteus vulgaris* (NCIM 2027), and one clinical MDR uropathogenic *Escherichia coli* isolate.

### Adaptive evolution protocol

**Fig. 1:**
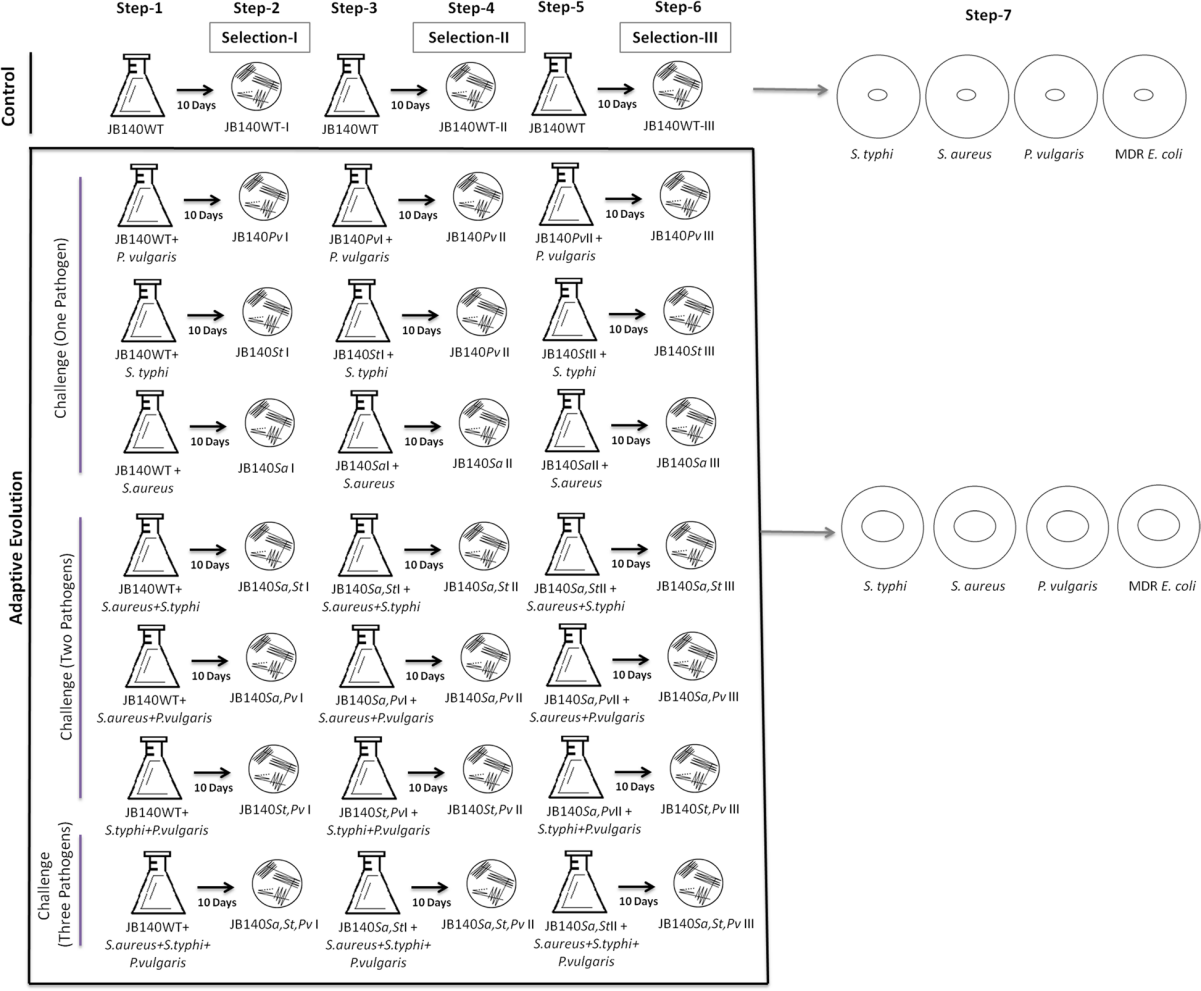
Adaptive evolution protocol (seven-steps) wherein a weak antibiotic-producing *Streptomyces* strain JB140 was challenged against bacterial pathogens

In the present adaptive laboratory evolution protocol, seven different competition experiments were performed in which a wild-type (WT) *Streptomyces* strain JB140 that exhibits very little antimicrobial activity was challenged against bacterial pathogens in different ways as depicted in Fig. 1. The first three (1st, 2nd, 3rd) experiments involved JB140^WT^ and one of the three target pathogens (a bi-culture competition consisting of JB140^WT^ and *P. vulgaris* or *S.* Typhi or *S. aureus*). The next three experiments (4th, 5th, 6th) involved JB140^WT^ and two target pathogens (a tri-culture competition consisting of JB140^WT^ and *S. aureus + S.* Typhi or *S. aureus + P. vulgaris* or *S.* Typhi *+ P. vulgaris*). The last experiment (7th) involved JB140^WT^ and three target pathogens (a quadri-culture competition consisting of JB140^WT^ and *S. aureus + S.* Typhi *+ P. vulgaris*). A mono-culture involving only JB140^WT^ served as an internal control. The seven experiments were performed separately by inoculating 50 mL of ISP2 broth medium in a 250-mL flask with a loopful of JB140^WT^ mycelia. After inoculation, the flasks were covered with a cotton plug and incubated at 37 ^o^C in a shaker incubator at 100 rpm for 48 h. After observing the visible growth of JB140^WT^, the flasks for bi-culture, tri-culture, and quadri-culture were inoculated accordingly with 100 μl (optical density at 600 nm = 0.50) of the target pathogen(s) and incubated at 37 ^o^C for 10 days as shown in Fig. 1 (step 1). The flask for mono-culture contained only JB140^WT^.

After this step, on the eleventh day, 100 μl from 10-day old mono-culture, bi-culture, tri-culture, and quadri-culture were spread on the fresh ISP2 agar medium and incubated at 37 ^o^C for 5 days to separate JB140^WT-^I (control), JB140^*Pv-*^I (bi-culture), JB140^*St-*^I (bi-culture), JB140^*Sa-*^I (bi-culture), JB140^*Sa,St-*^I (tri-culture), JB140^*Sa,Pv-*^I (tri-culture), JB140^*St,Pv-*^I (tri-culture), and JB140^*Sa,St,Pv-*^I (quadri-culture) from their respective flasks (step 2) (Fig. 1). After purification, the next cycle of adaptation-selection was repeated by inoculating purified wild-type and evolved mutants from step 2 into the fresh ISP2 medium. It is important to mention here that the flasks in the successive steps were introduced with freshly grown pathogen/s from their respective stocks. The inoculated flasks were incubated for another 10 days (step 3; II cycle). After incubation, the mixed cultures were purified to obtain JB140^WT-^II (control), JB140^*Pv-*^II (bi-culture), JB140^*St-*^II (bi-culture), JB140^*Sa-*^II (bi-culture), JB140^*Sa,St-*^II (tri-culture), JB140^*Sa,Pv-*^II (tri-culture), JB140^*St,Pv-*^II (tri-culture), and JB140^*Sa,St,Pv-*^II (quadri-culture) from their respective flasks (step 4). The adaptive evolution cycle (III cycle) was again repeated to start a new adaptation-selection cycle in steps 5 and 6 to finally receive the wild-type JB140^WT-^III and evolved mutants JB140^*Pv-*^III, JB140^*St-*^III, JB140^*Sa-*^III, JB140^*Sa,St-*^III, JB140^*Sa,Pv-*^III, JB140^*St,Pv-*^III, and JB140^*Sa,St,Pv-*^III.

The protocol described here, consisting of six main steps that took ~ 45 days to complete (Fig. 1). The wild-type and evolved isolates were preserved in the glycerol stocks (20%v/v) at -40 ^o^C. After each cycle of adaption-selection, the antimicrobial activities of the wild-type and evolved mutants were assayed against pathogenic *Salmonella* Typhi (NCIM 2051), *Staphylococcus aureus* (NCIM 2079), and *Proteus vulgaris* (NCIM 2027) using the agar well diffusion method. The antimicrobial activity of the mutants purified after the third cycle was also tested against a clinical MDR uropathogenic *Escherichia coli* (UPEC 1021) isolate in step 7. The detailed prototype of the present method has been also provided in supplementary data (Fig. S1 and Fig. S2).

### DNA extraction, sequencing, and phylogenetic analysis

The wild-type isolate JB140 and its evolved mutants JB140^*Pv-*^III, JB140^*St-*^III, JB140^*Sa-*^III, JB140^*Sa,St-*^III, JB140^*Sa,Pv-*^III, JB140^*St,Pv-*^III, and JB140^*Sa,St,Pv-*^III were subjected to 16SrRNA gene sequence analysis [24] for their precise identification. The genomic DNA of each isolate was extracted using a method of Li et al. [25]. 16SrRNA gene from each was amplified using a primer pair 27F 5′AGA GTT TGA TCC TGG CTC AG3′ and 1492R 5′GGT TAC CTT GTT ACG ACT T3′. The reaction started with an initial denaturation at 95 ^o^C for 5 min, followed by 30 cycles of DNA denaturation at 95 ^o^C for 1 min, primer-annealing at 44 ^o^C for 1 min and extension cycle at 72 ^o^C for 1.5 min, with a final extension at 72 ^o^C for 10 min. PCR-amplicons were visualized in 2% agarose gel electrophoresis and subsequently revealed with ethidium bromide staining. The amplified 16SrDNA gene product was sequenced using Sanger dideoxy method. Manual sequence edition, alignment, and contig assembly were performed using Vector NTI v10 software package. Sequencing results were analyzed for chimeras using DECIPHER v1.4.0 program [26]. The 16SrRNA gene sequences were compared with the GenBank/EMBL/DDBJ databases by using the BLASTN [27] search program. After pair wise alignment using CLUSTAL_X program v1.8 [28], the phylogenetic tree of the wild-type isolate JB140 was constructed by neighbor-joining [29] method using MEGA X [30]. The evolutionary distance was computed using the Kimura 2-parameter method [31]. The stability of relationships was assessed by performing bootstrap analyses [32] of the neighbor-joining data based on 1000 re-samplings.

### Chemical characterization

To characterize the proteinaceous nature of antimicrobial metabolites produced by the wild-type *Streptomyces* strain and its evolved mutants, methanolic extract of JB140^*Sa,St,Pv*^ (purified after III^rd^ cycle) was investigated against proteolytic enzymes trypsin and proteinase K (Sigma). The methanolic extract of JB140^*Sa,St,Pv*^ was mixed with trypsin and proteinase K at final concentrations of 0.5 and 10 mg/ml, respectively. The reaction was incubated at 37 ^o^C for 24 h. The temperature sensitivity was also examined by heating the methanolic extracts prepared from the 9-day grown cultivation broth of JB140^WT^ and JB140^*Pv*^, JB140^*St*^, JB140^*Sa*^, JB140^*Sa,St*^, JB140^*Sa,Pv*^, JB140^*St,Pv*^, and JB140^*Sa,St,Pv*^ to 90 ^o^C for 1 h. Subsequently, the reactions were tested against the bacterial pathogens. The methanolic extracts prepared from the wild-type and evolved *Streptomyces* mutants were also analyzed by SDS-PAGE.

### RAPD profiling

Random amplified polymorphic DNA (RAPD) analysis of the wild-type strain JB140 and its evolved mutants was performed by using random primers (Operon Technologies, USA); RAPD-1 5′AAGAGCCCGT3′, RAPD-2 5′GTTTCCGCCC 3′ and RAPD-3 5′GAGGCCCTTC 3′. These three RAPD primers were used individually or in combination (all three) in separate PCR amplification cycles. The reaction started with an initial denaturation at 95 ^o^C for 5 min, followed by 40 cycles of DNA denaturation at 95 ^o^C for 1 min, primer-annealing at 32 ^o^C for 1 min, and extension cycle at 72 ^o^C for 1 min, with a final extension at 72 ^o^C for 10 min. PCR-amplicons were visualized in 2% agarose gel electrophoresis after ethidium bromide staining. The detailed prototype of the present method has been also provided in supplementary data (Fig. S2).

## Results

### Isolation and characterization of JB140

**Fig. 2:**
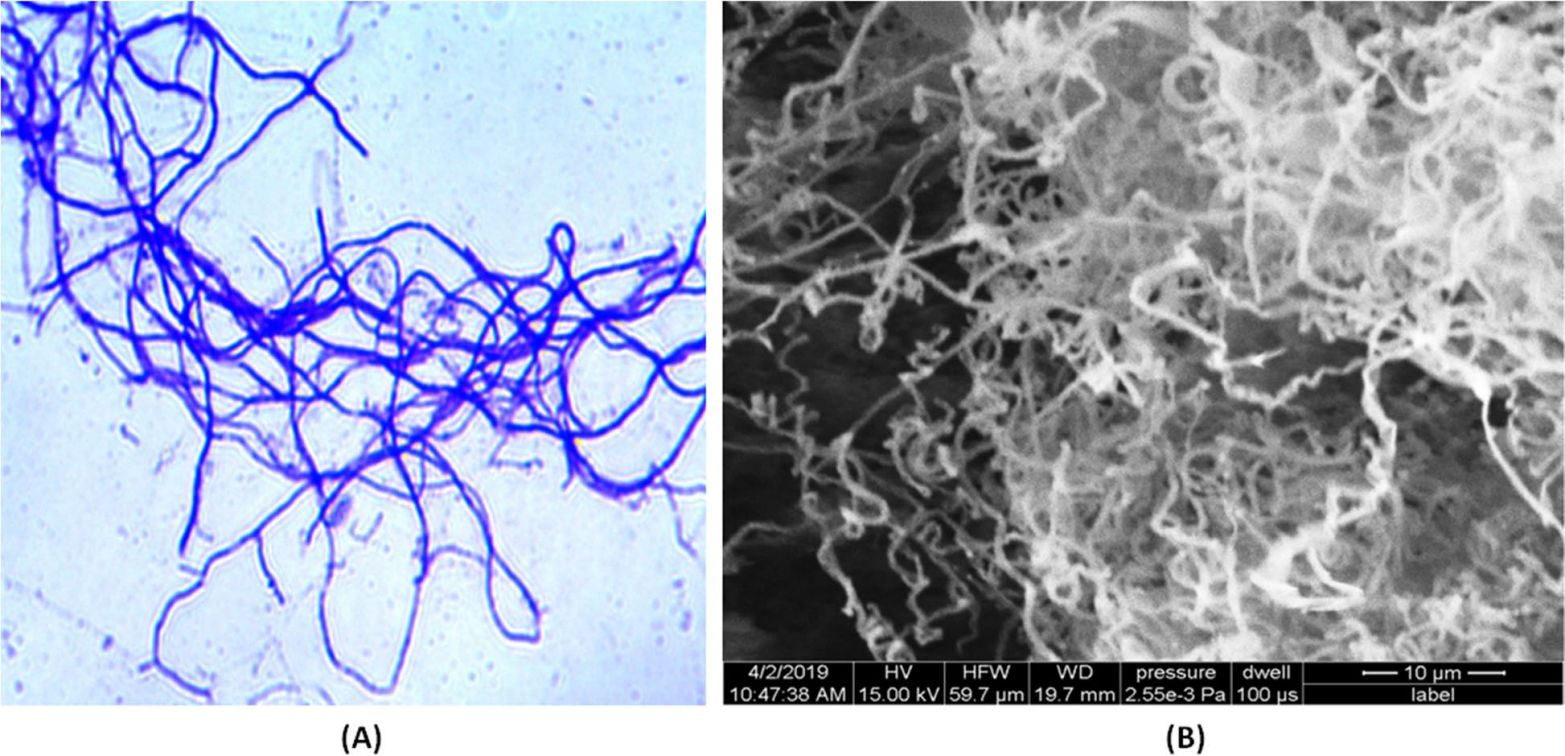
Photomicrograph (× 1000) of mycelia (**A**) and scanning electron micrograph of spores (**B**) of *Streptomyces* isolate JB140

**Fig. 3:**
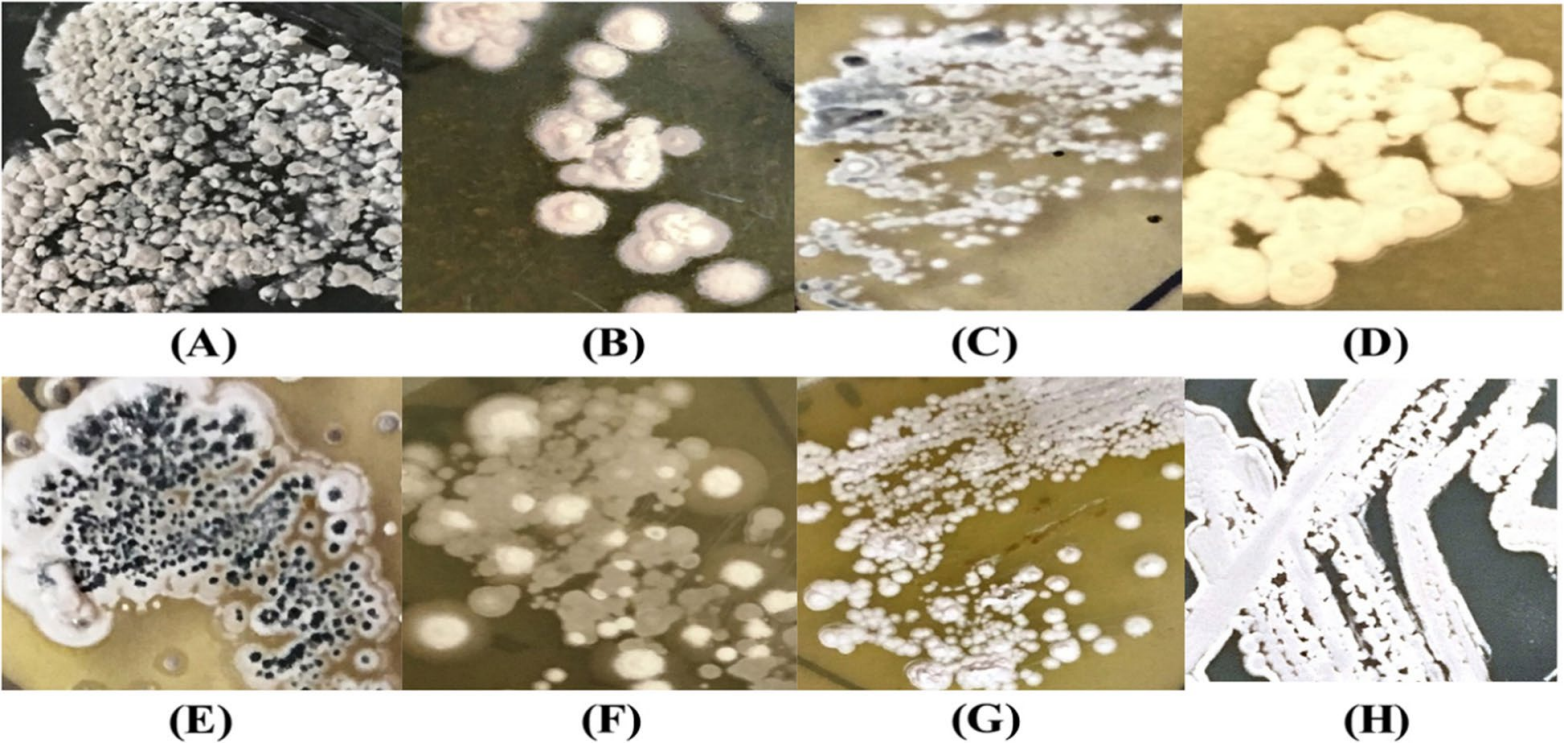
Colonial appearance of the isolate JB140 on **A** modified actinomycetes specific (MAS) medium, **B** tryptone yeast extract agar (ISP1), **C** yeast extract maltose agar (ISP2), **D** oatmeal agar (ISP3), **E** inorganic salt starch agar (ISP4), **F** glycerol asparagine agar (ISP5), **G** peptone yeast extract iron agar (ISP6), **H** tyrosine agar (ISP7)

Based on characteristic colonial morphology, notably the ability to form aerial hypha and substrate mycelia, earthy smell and chalky appearance of the mature colony, darker in the center and lighter farther, irregular, fuzzy edge, pigmented, strongly adhered and leathery texture, the isolate was putatively identified as actinobacteria. The Gram-positive actinobacterial isolate JB140 showed a typical mycelial structure in Gram’s staining (Fig. 2A). The scanning electron micrograph of the isolate JB140 revealed that it has a long looped retinaculiaperti spore chain with a smooth surface (Fig. 2B). The isolate exhibited well-developed aerial and substrate mycelium on various ISP media (Fig. 3).

### 16SrRNA gene sequence analysis and phylogeny

**Fig. 4:**
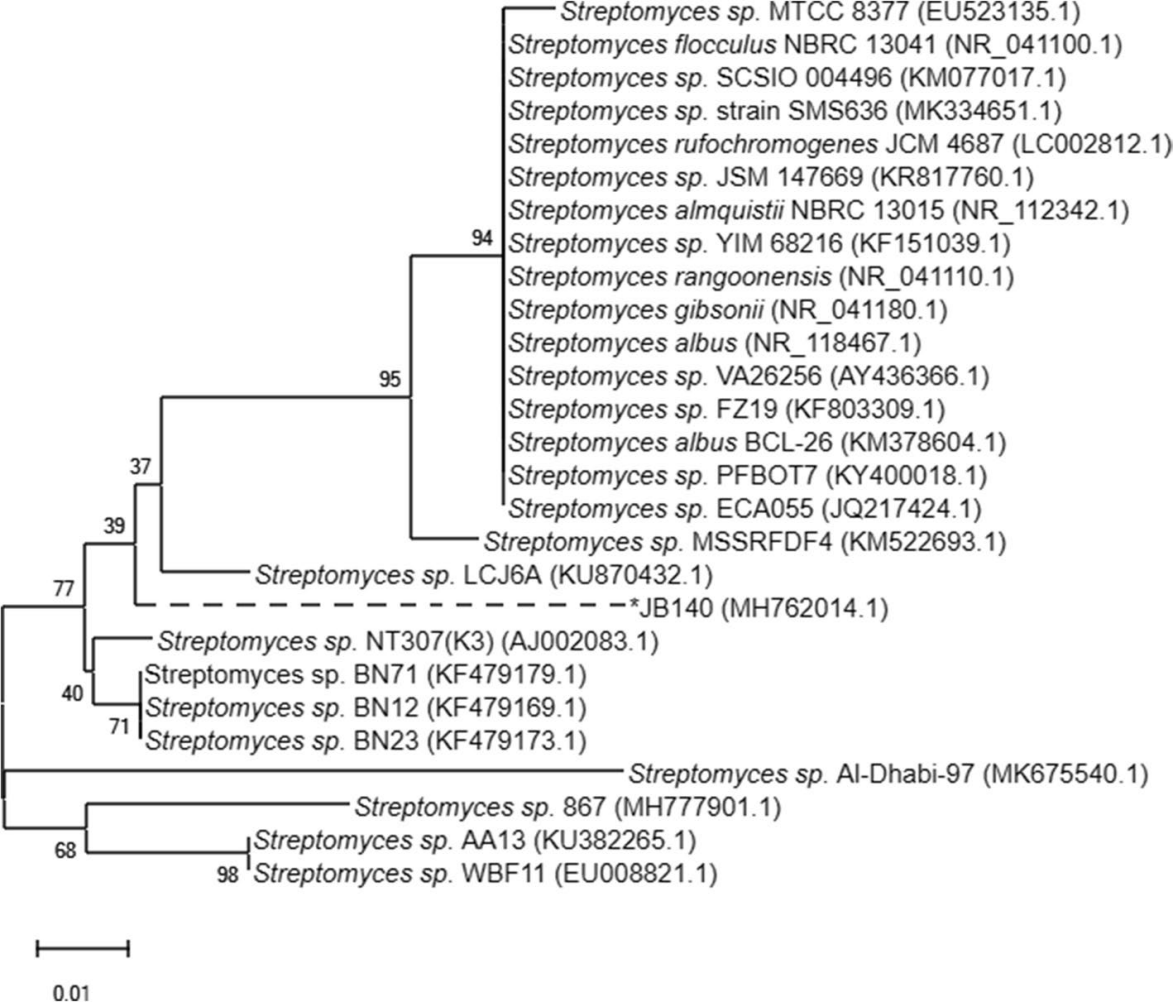
Evolutionary relationship of JB140 was inferred using the neighbor-joining method. The tree is drawn to scale, with branch lengths in the same units as those of the evolutionary distances used to infer the phylogenetic tree. The evolutionary distances were computed using the Kimura 2-parameter method. The rate variation among sites was modeled with a gamma distribution (shape parameter = 2). Evolutionary analysis was conducted in MEGA X

A comparison of the 16SrRNA gene sequence of the isolate JB140 was made against the available sequences in the GenBank database. It revealed homology of greater than 98% to the members of genera *Streptomyces*. It displayed 97.32 to 98.21% 16SrRNA gene sequence similarities to the closest type strain *Streptomyces* with 98.21% identity to the nearest neighbor *Streptomyces* sp. BN71 (KF479179). The evolutionary relationship of the strain JB140 (MK855143) and the other 26 *Streptomyces* spp. has been provided in a phylogenetic tree in Fig. 4.

### Assessment of antimicrobial activity

**Fig. 5:**
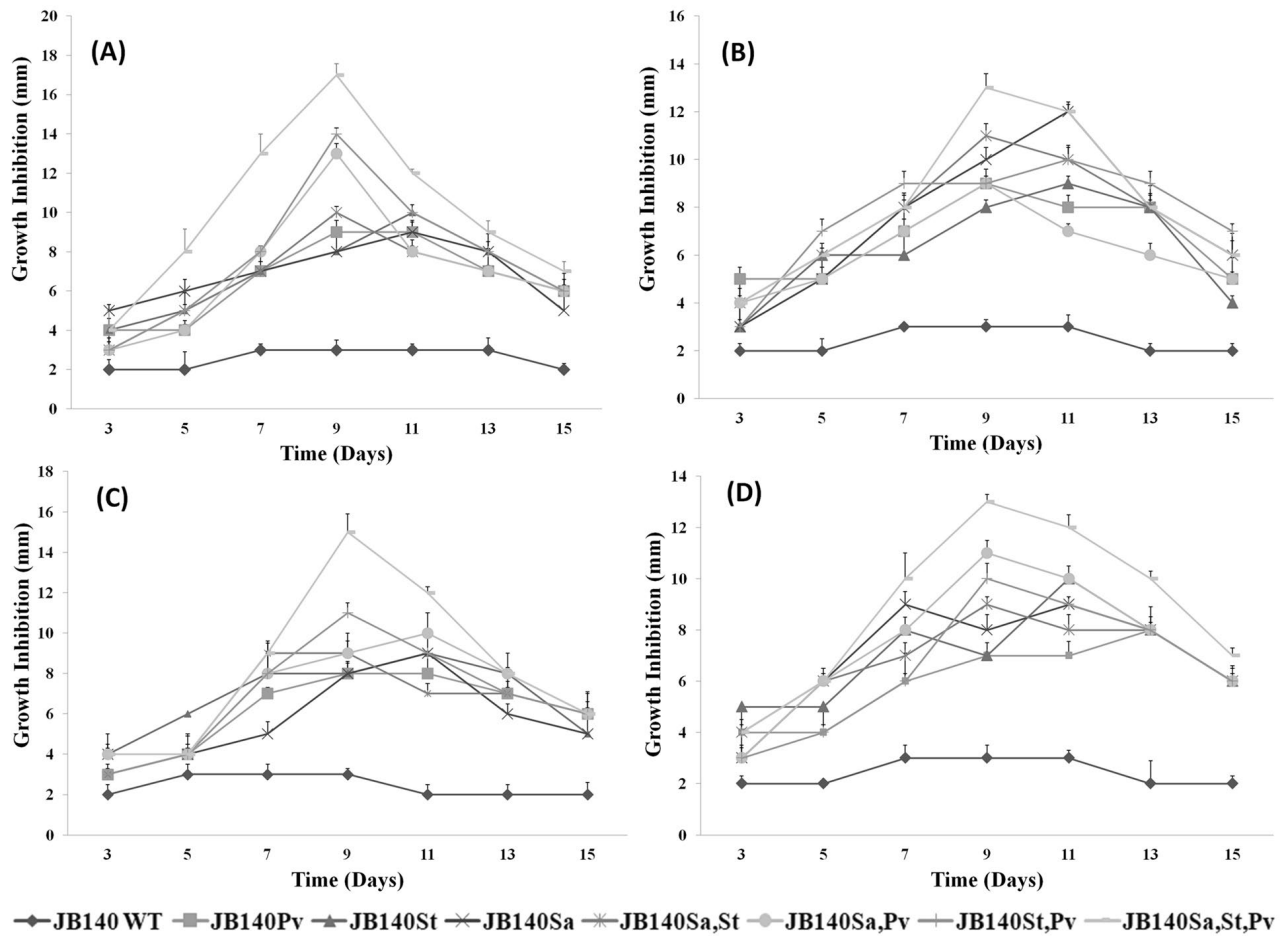
Antimicrobial activity of JB140^WT^ strain and evolved JB140^*Pv*^, JB140^*St*^, JB140^*Sa*^, JB140^*Sa,St*^, JB140^*Sa,Pv*^, JB140^*St,Pv*^, and JB140^*Sa,St,Pv*^ mutants against *Salmonella* Typhi (**A**), *Staphylococcus aureus* (**B**), *Proteus vulgaris* (**C**) and a clinical MDR uropathogenic *Escherichia coli* (UPEC 1021) (**D**). Values represent the mean ± SD for three replications

The isolate JB140 was analyzed for its antibacterial activity in primary and secondary screening against *Proteus vulgaris*, *Salmonella* Typhi, *Staphylococcus aureus*, and a clinical MDR UPEC. The antimicrobial activity of the isolate was estimated by measuring the diameter of the clear zone of growth inhibition (in mm scale). The methanolic extract of the isolate JB140 exhibited very little antimicrobial activity against the pathogenic bacteria tested as the inhibition zone of only 2–3 mm diameter was observed (Fig. 5).

### Adaptive evolution of *Streptomyces* JB140

Using adaptive laboratory evolution protocol, *Streptomyces* strain JB140^WT^ competed against three different pathogens in the bi-, tri-, and quadri-culture experiments (Fig. 1). In ~ 45-day-long competition experiments, after the third (III) cycle of adaptation-selection (after three serial passages of 10 days each) seven mutants were recovered and purified. After each cycle of adaption-selection, the antimicrobial activity of the wild-type and evolved mutants was assayed using the agar well diffusion method. An increased antimicrobial activity of the evolved mutants was noticed only after the third (III) cycle of adaptation-selection (Fig. 5). Whereas, after the first (I) and second (II) cycles of serial adaptation-selection, very little or no antimicrobial activity was detected in the evolved mutants. These unique mutants, purified after the third (III) cycle of serial adaptation-selection were designated as JB140^*Pv*^, JB140^*St*^, JB140^*Sa*^, and JB140^*Sa,St*^, JB140^*Sa,Pv*^, JB140^*St,Pv*^, and JB140^*Sa,St,Pv*^. All seven mutants produced stable growth inhibitory activity against the tested pathogens. To ascertain that the stimulation of the production of antimicrobials in JB140 mutants is due to three serial adaptive-selection cycles wherein they were repeatedly exposed to the pathogens, JB140WT (a mono-culture experiment without a competing pathogen) was also inoculated using the same procedure. After 45 days (after III cycle in step 7), JB140^WT^ was also assessed for its antimicrobial activity against the tested pathogens. The antimicrobial activity was observed to be similar as exhibited by its wild-type counterpart (Fig. 5). Contrary to this, the growth inhibitory effect of the evolved mutants against bacterial pathogens was observed to be from 9 mm to 14 mm in diameter. The highest antimicrobial activity of the evolved mutants was registered after 9–11 days duration and after that interval, it declined. Mutants JB140^*St,Pv*^ and JB140^*Sa,St*^ demonstrated the highest activity against *S.* Typhi (Fig. 5A) while mutants JB140^*Sa*^, JB140^*Sa,St*^, and JB140^*Sa,St,Pv*^ were noticed to be highly active against *S. aureus* (Fig. 5B). The highest antimicrobial response was observed by JB140^*St,Pv*^ and JB140^*Sa,St,Pv*^ mutants against *P. vulgaris*, (Fig. 5C). Interestingly, all the evolved mutants were also found to exhibit antimicrobial activity against a clinical MDR uropathogenic *Escherichia coli* (UPEC 1021) and the highest activity was registered for the mutants JB140^*St,Pv*^ and JB140^*Sa,St,Pv*^ (Fig. 5D). Furthermore, among seven different competition experiments, the highest antimicrobial activity was observed with the mutants that were evolved against the three pathogens (quadri-culture competition) (Fig. 5). The overall observations from the present study suggest that the growth inhibitory effect of the evolved mutants against the tested pathogens including clinical MDR uropathogenic *E. coli* appeared after three serial passages of adaptation-selection only and thus induced antimicrobial activity was not associated with JB140^WT^ parental strain. A similar growth inhibitory effect of the evolved mutants was also detected against nonpathogenic bacterial strains namely *Escherichia coli* (K-12 W3110) and *Bacillus subtilis* (NCIM 2920) (data not shown). Intriguingly, the whole process of laboratory-based adaptive evolution was reproducible and we could be able to replicate the results when the strategy was tested 3 months apart.

### Characterization of evolved mutants

**Fig. 6:**
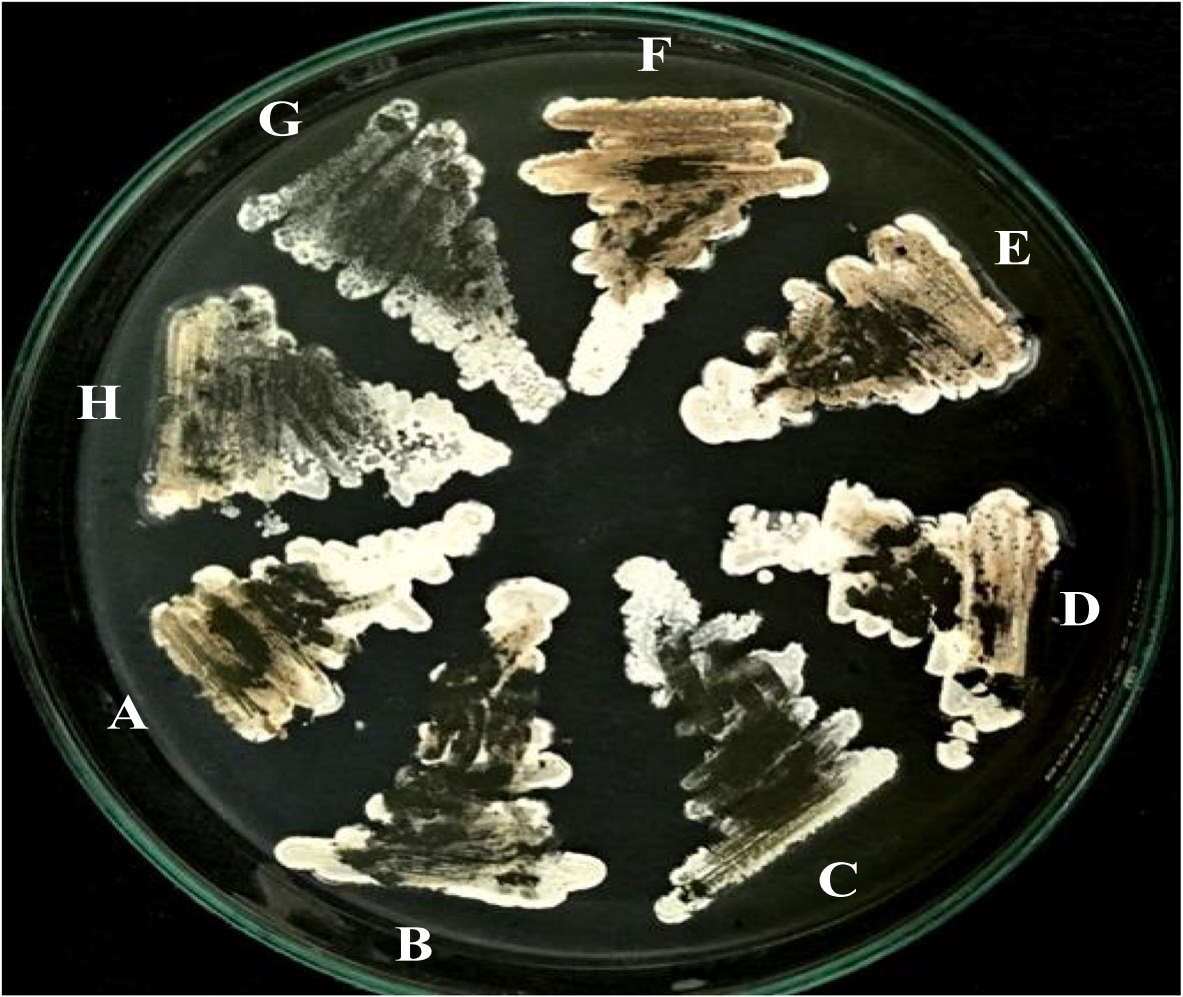
Phenotypic appearance of *Streptomyces* strain JB140^WT^ (**A**) and its evolved mutants JB140^*St*^ (**B**), JB140^*Sa*^ (**C**), JB140^*Pv*^ (**D**), JB140^*Sa,St*^ (**E**), JB140^*Sa,Pv*^ (**F**), JB140^*St,Pv*^ (**G**), and JB140^*Sa,St,Pv*^ (**H**) grown on ISP2 medium

**Fig. 7:**
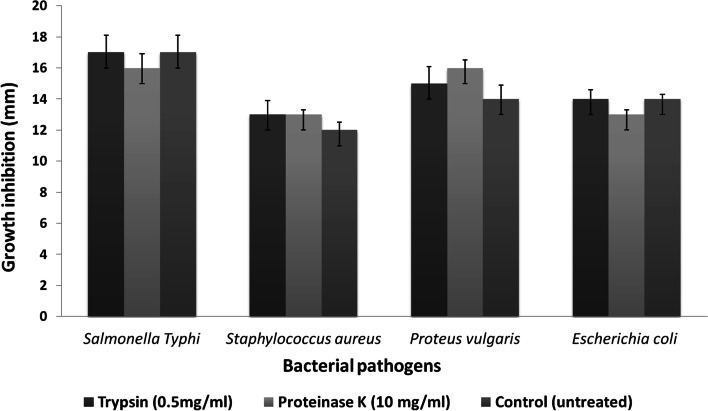
Antimicrobial activity of JB140^*Sa,St,Pv*^ against *Salmonella* Typhi, *Staphylococcus aureus*, *Proteus vulgaris*, and a clinical MDR uropathogenic *Escherichia coli* isolate after treatment with 0.5 mg/ml trypsin and 10 mg/ml proteinase K. Values represent the mean ± SD for three replications

**Fig. 8:**
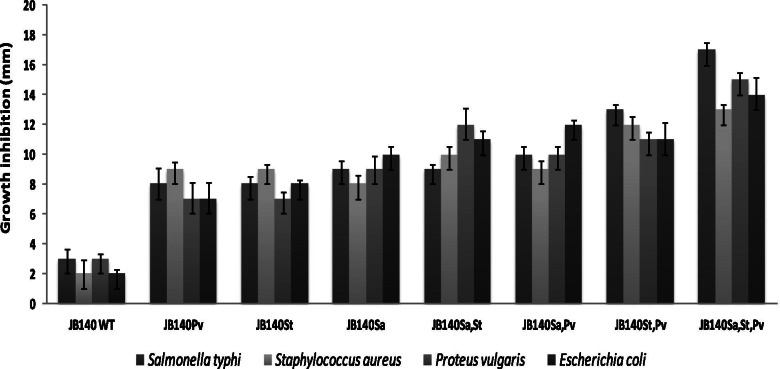
The antimicrobial response of JB140^WT^ and evolved mutants JB140^*Pv*^, JB140^*St*^, JB140^*Sa*^, JB140^*Sa,St*^, JB140^*Sa,Pv*^, JB140^*St,Pv*^, and JB140^*Sa,St,Pv*^ against *Salmonella* Typhi, *Staphylococcus aureus*, *Proteus vulgaris,* and a clinical MDR uropathogenic *Escherichia coli* isolate after heat treatment at 90 ^o^C. The methanolic extracts were prepared from 9-day grown cultivation broth

To confirm that the evolved mutants JB140^*Pv*^, JB140^*St*^, JB140^*Sa*^, JB140^*Sa,St*^, JB140^*Sa,Pv*^, JB140^*St,Pv*^, and JB140^*Sa,St,Pv*^ are indeed the true clone of *Streptomyces* JB140^WT^ and are not contaminants, the identity of these were confirmed using 16SrRNA gene sequence analyses. All seven mutants JB140^*Pv*^ (MK850143), JB140^*St*^ (MK852169), JB140^*Sa*^ (MK852177), JB140^*Sa,St*^ (MK852179), JB140^*Sa,Pv*^ (MK852175), JB140^*St,Pv*^ (MK852170), and JB140^*Sa,St,Pv*^ (MK852176) were confirmed for their identity as *Streptomyces*, similar to their parental wild-type JB140^WT^ (MK855143). Sequence comparison showed that all mutants were true clones of the JB140^WT^ strain (data not shown). The phenotypic characteristics of the evolved mutants were also observed to be the same as their parental JB140^WT^ strain (Fig. 6). The sensitivity of methanolic extract prepared from the evolved mutant JB140^*Sa,St,Pv*^ was unaffected to proteolytic enzymes trypsin and proteinase K. The antimicrobial effect of JB140^*Sa,St,Pv*^ against all the tested bacterial pathogens was prominent (Fig. 7). The antimicrobial response of the JB140^WT^ and JB140^*Pv*^, JB140^*St*^, JB140^*Sa*^, JB140^*Sa,St*^, JB140^*Sa,Pv*^, JB140^*St,Pv*^, and JB140^*Sa,St,Pv*^ mutants were also observed to be retained at 90 ^o^C (Fig. 8). Moreover, no protein or polypeptide band was observed when the wild-type JB140 and its mutants were analyzed by SDS-PAGE.

### RAPD profiling

**Fig. 9:**
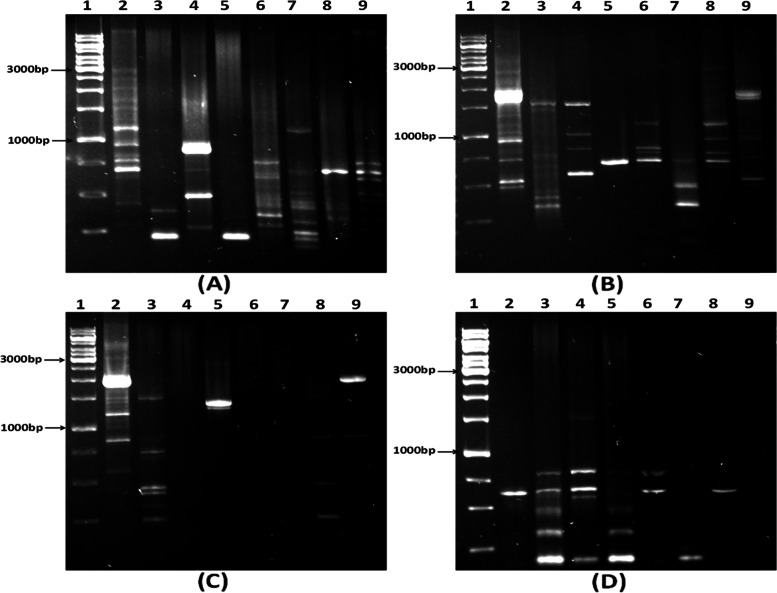
Detection of genetic variation between JB140^WT^ and evolved mutants using RAPD primer 1 (**A**), RAPD primer 2 (**B**), RAPD primer 3 (**C**), and RAPD primers 1,2,3 (**D**). Lanes 1-1 kb DNA ladder, 2-JB140^WT^, 3-JB140^*Pv*^, 4-JB140^*St*^, 5-JB140^*Sa*^, 6-JB140^*Sa,St*^, 7-JB140^*Sa,Pv*^, 8-JB140^*St,Pv*^, and 9-JB140^*Sa,St,Pv*^

In support of adaptive evolution competition experiments and to get evidence of the evolution in actinobacterial JB140^WT^, random amplified polymorphic DNA analysis (RAPD) was performed. Genomic DNA of JB140^WT^ and JB140^*Pv*^, JB140^*St*^, JB140^*Sa*^, JB140^*Sa,St*^, JB140^*Sa,Pv*^, JB140^*St,Pv*^, and JB140^*Sa,St,Pv*^ were amplified by using three RAPD primers (1, 2, and 3) and compared. The genomic DNA was amplified in separate amplification cycles using RAPD-1, RAPD-2, and RAPD-3 primers individually or in combination (all three). A large number of DNA bands were visualized in the RAPD profiles obtained from the wild-type strain and its mutants that represented significant genetic variations between them (Fig. 9). In a few instances, the RAPD-3 primer was not able to exhibit significant polymorphism between the evolved mutants (Fig. 9C). Similarly, we could not detect any polymorphic DNA in the case of JB140^*Sa,St,Pv*^ with RAPD-1,2,3 primers (Fig. 9D). Nevertheless, detectable differences were observed in the DNA profiles from the wild-type strain JB140 and its evolved mutants using RAPD-1 (Fig. 9A), RAPD-2 (Fig. 9B), and RAPD-1,2,3 primers (Fig. 9D).

## Discussion

Many attempts have been made to develop laboratory methods to stimulate the production of novel secondary metabolites [33–38]. One of these methods includes adaptive laboratory evolution that has been successfully used to augment the production of antimicrobial molecules [39–43]. The adaptive laboratory evolution is a strategy in which a parent strain is serially passed against a certain selection pressure for few generations to promote adaptation to a new prevailing niche. The strategy has helped to increase our current understanding of natural laws of evolution that may further provide convincing solutions to the rising problems such as multidrug resistance [44–46]. Over a century ago, experiments involving adaptive laboratory evolution were originally performed [47–49]. However, in the present time, there has been an increase in the number of such experiments that involve microorganisms particularly yeast and bacteria to identify improved phenotypes and genotypes [40, 50–53]. A well-described example of this strategy is the adaptive laboratory evolution of antibiotic resistance in *E. coli* lineages under mild selection that displays a selective growth advantage independently of the acquired level of antibiotic resistance [53]. Moreover, the strategy can also assist to understand fundamental evolutionary principles that regulate detrimental adaptations [54]. Several other examples are also available in the literature that well describe this strategy such as the evolution of *E. coli* growing in glucose minimal medium to adapt in the media containing glycerol and lactate [55] and alteration in the productivity of industrially important chemicals [56, 57]. Furthermore, the strategy has been also tested in different assays to observe ethanol tolerance [58–60], gene regulation in response to osmotic stress in *Escherichia coli* [61], and *Streptomyces* [62], activation of glycerol metabolism in *Xanthomonas campestris* [63] and enhanced extracellular electron transfer in *Geobacter sulfurreducens* [64]. It is important to note that the change in ecological niche causes the adaptive evolutionary process to occur in microbes, driven by genetic changes such as gain or loss, differential expression of existing genes [64–68]. We applied this strategy in the present work to study the importance of serial passages over time in the adaptive evolution of a *Streptomyces* wild-type strain with very little antimicrobial activity into the *Streptomyces* phenotypes (mutants) exhibiting broad-spectrum antimicrobial activity.

One of the main findings of the present work is that we were able to identify mutants that produced an increased amount of antimicrobials to inhibit the growth of bacterial pathogens effectively, including a clinical MDR uropathogenic *Escherichia coli* (UPEC 1021)*,* as compared to their parental wild-type counterpart. Interestingly, when the challenge was posed by more than one pathogen in the assays, the stronger antimicrobial response was elicited by the producer strain. Among seven different competition experiments, the highest antibiotic response was registered with the mutants that were adaptively evolved against the three pathogens (quadri-culture competition). Thus, it could be stated that the evolved unique phenotypes arose only after three serial adaptation-selection cycles and not after initial, short-term exposure to the selection pressure. Other studies have been also performed in which augmented antibiotic production was observed wherein microorganisms were grown in a co-culture [13–15, 17, 69, 70]. However, in the present study, the wild-type strain was not detected to exhibit antimicrobial potential until it was exposed to bacterial pathogens and serially passed for three successive cycles of 15 days each. And importantly, the requirement of repeated adaptation-selection cycles was crucial for the producer strain to exhibit stimulated production of antimicrobials against the target pathogens. The pathogens tested in the present study never faced serial passages. It may be speculated that during this period, competitive exclusion [71, 72] provoked the wild-type *Streptomyces* strain to synthesize chemical molecules to resist the challenge posed by the competing pathogen. When the wild-type *Streptomyces* strain was exposed to more than one pathogen, a higher antimicrobial response was evident. Although the type of chemical molecules (secondary metabolites) stimulated and the precise regulatory mechanism of transforming a wild-type *Streptomyces* strain exhibiting little antimicrobial effect into the phenotype with strong antimicrobial effect using this strategy is yet to be confirmed. Nevertheless, a simple procedure described in the present study certainly could assist in the discovery of yet undiscovered natural metabolites.

The antimicrobial response of the JB140^*Sa,St,Pv*^ mutant was insensitive to proteolytic enzymes trypsin and proteinase K. Moreover, the stability of the crude extracts at high temperature and the absence of protein bands in SDS-PAGE indicates that the antimicrobial metabolites produced by JB140^WT^ and JB140^*Pv*^, JB140^*St*^, JB140^*Sa*^, JB140^*Sa,St*^, JB140^*Sa,Pv*^, JB140^*St,Pv*^, and JB140^*Sa,St,Pv*^ might not be proteinaceous. We could collect molecular evidence for the adaptive laboratory evolution by comparing RAPD profiles of the wild-type *Streptomyces* and its evolved mutants. To detect these genetic variations, three RAPD primers were tested individually and in combinations. In principle, there is no upper limit to the number of RAPD primers that can be used to detect the DNA polymorphism. It is also reasonable to believe that if “n” primers are used in an RAPD assay, a total of “xn2” bands should be generated provided no factors are limiting the RAPD reaction. However, as can be seen in Fig. 9D, wherein RAPD-1,2,3 primers were used in combination, the average number of polymorphic DNA does not increase expectedly and remained constant to some extent. This is consistent with the results of Hu et al. [73] and Säull [74], who compared single and multiple-primer RAPD reactions and observed that RAPD assays involving multiple primers, as compared to a single-primer, yield fewer bands on average. It was speculated that competition between multiple-RAPD-primers in the reaction operates in such a way that after PCR amplification merely a subset of DNA polymorphic bands is detected [75, 76]. Nevertheless, in the present study, RAPD assays yielded significant polymorphism between the wild-type strain and its evolved mutants. However, the exact mutational events that occurred during this evolution are yet unknown. In this regard, future work will be important to reveal the underlying mechanism responsible for the laboratory-based adaptive evolution strategy. To apply and test this method on other systems, a detailed prototype has been also provided in the supplementary data of the manuscript (Fig. S1 and Fig. S2).

## Conclusion

A wild-type *Streptomyces* strain exhibiting very little antimicrobial activity, when competed against bacterial pathogens using adaptive evolution protocol, was transformed into the unique mutant phenotype with increased antimicrobial profiles. These improved phenotypes were stable and acquired the ability to constitutively exhibit increased antimicrobial activity against various bacterial pathogens as compared to their parental wild-type counterpart. Moreover, the molecular evidence of the genetic variation collected using RAPD profiling revealed significant polymorphism in the evolved mutants. The constitutive overproduction of the antimicrobials (non-proteinaceous) in the mutants was possibly due to the expression of genes that were silent or not expressed in the wild-type parent. Conclusively, the present study signifies that adaptive laboratory evolution is an efficient tool to select improved phenotypes that can overproduce target-dependent bioactive metabolites. The present study also provides a framework to design more improved selection methods to find out the possible solutions to the issues posed by MDR pathogens and provide much-needed novel and alternative antimicrobial therapy.

## Supplementary Information

**Additional file 1.** Method details. Supplementary Fig. S1 A seven-step adaptive evolution protocol wherein a wild-type producer strain is competed against bacterial pathogens to stimulate antibiotic production. Supplementary Fig. S2 RAPD analysis to detect genetic variation between wild-type producer and evolved mutants using RAPD primers.

## Data Availability

The authors declare that all data generated or analyzed in this study are included in the article.

## Abbreviations

WT: Wild type
MDR: Multidrug-resistant
RAPD: Random amplified polymorphic DNA
St: *Salmonella* Typhi
Sa: *Staphylococcus aureus*
Pv: *Proteus vulgaris*
MAS: Modified Actinomycete Selective
SEM: Scanning electron microscopy
ISP: International *Streptomyces* Project
UPEC: Uropathogenic *Escherichia coli*
